# Hydroxychloroquine and Azithromycin Treatment of Hospitalized Patients Infected with SARS-CoV-2 in Senegal from March to October 2020

**DOI:** 10.3390/jcm10132954

**Published:** 2021-06-30

**Authors:** Fabien Taieb, Khardiata Diallo Mbaye, Billo Tall, Ndèye Aïssatou Lakhe, Cheikh Talla, Daouda Thioub, Amadou Moustapha Ndoye, Daye Ka, Aboubacry Gaye, Viviane Marie-Pierre Cissé Diallo, Ndongo Dia, Pape Samba Ba, Mamadou Cissé, Moustapha Diop, Cheikh Tidiane Diagne, Louise Fortes, Mamadou Diop, Ndèye Maguette Fall, Fatoumata Diène Sarr, Margarite Diatta, Mamadou Aliou Barry, Aboubakar Sidikh Badiane, Abdoulaye Seck, Philippe Dubrous, Ousmane Faye, Inès Vigan-Womas, Cheikh Loucoubar, Amadou Alpha Sall, Moussa Seydi

**Affiliations:** 1 Institut Pasteur de Dakar, Dakar BP 220, Senegal; billo.tall@pasteur.sn (B.T.); ctalla@pasteur.sn (C.T.); amadoumoustapha.ndoye@pasteur.sn (A.M.N.); aboubacry.gaye@pasteur.sn (A.G.); ndongo.dia@pasteur.sn (N.D.); mamadou.cisse@pasteur.sn (M.C.); CheikhTidiane.DIAGNE@pasteur.sn (C.T.D.); mamadou.diop@pasteur.sn (M.D.); fatoumata.sarr@pasteur.sn (F.D.S.); Marguerite.diatta@pasteur.sn (M.D.); aliou.barry@pasteur.sn (M.A.B.); ablayseck@gmail.com (A.S.); Philippe.dubrous@pasteur.sn (P.D.); Ousmane.faye@pasteur.sn (O.F.); Ines.vigan-womas@pasteur.sn (I.V.-W.); cheikh.loucoubar@pasteur.sn (C.L.); Amadou.sall@pasteur.sn (A.A.S.); 2 Service des Maladies Infectieuses et Tropicales, De Fann University Hospital Center (CNHU FANN), Dakar BP 5035, Senegal; diallokhardiata@gmail.com (K.D.M.); aissatoulakhe@gmail.com (N.A.L.); dave11690@gmail.com (D.T.); dayeka10@gmail.com (D.K.); vivich6@gmail.com (V.M.-P.C.D.); louisefortes@gmail.com (L.F.); maguifall4@gmail.com (N.M.F.); aboubakrbadiane@yahoo.fr (A.S.B.); seydim@u.washington.edu (M.S.); 3 Hôpital Principal de Dakar, Dakar BP 220, Senegal; basambasn2000@yahoo.fr (P.S.B.); mouztaphandm@gmail.com (M.D.); 4 Centre de Traitement des épidémies de l’Hôpital Dalal Jamm, Dakar BP 19.001, Senegal; 5 Hôpital de Diamnadio, Centre de Traitement des épidémies de l’Hôpital de Diamnadio, Bargny BP 204, Senegal; 6 Fann University Hospital, Centre de Traitement des épidémies de Touba, Touba 22300, Senegal

**Keywords:** SARS-CoV-2, hydroxychloroquine, azithromycin, treatment, Senegal

## Abstract

As of today, little data is available on COVID-19 in African countries, where the case management relied mainly on a treatment by association between hydroxychloroquine (HCQ) and azithromycin (AZM). This study aimed to understand the main clinical outcomes of COVID-19 hospitalized patients in Senegal from March to October 20202. We described the clinical characteristics of patients and analysed clinical status (alive and discharged versus hospitalized or died) at 15 days after Isolation and Treatment Centres (ITC) admission among adult patients who received HCQ plus AZM and those who did not receive this combination. A total of 926 patients were included in this analysis. Six hundred seventy-four (674) (72.8%) patients received a combination of HCQ and AZM. Results showed that the proportion of patient discharge at D15 was significantly higher for patients receiving HCQ plus AZM (OR: 1.63, IC 95% (1.09–2.43)). Factors associated with a lower proportion of patients discharged alive were: age ≥ 60 years (OR: 0.55, IC 95% (0.36–0.83)), having of at least one pre-existing disorder (OR: 0.61, IC 95% (0.42–0.90)), and a high clinical risk at admission following NEWS score (OR: 0.49, IC 95% (0.28–0.83)). Few side effects were reported including 2 cases of cardiac rhythmic disorders in the HCQ and AZM group versus 13 in without HCQ + AZM. An improvement of clinical status at 15 days was found for patients exposed to HCQ plus AZM combination.

## 1. Introduction

On 31 December 2019, the WHO China Country Office was informed of cases of pneumonia of unknown aetiology detected in Wuhan City, Hubei Province of China [1]. The COVID-19 was declared as a pandemic by WHO [2]. From the middle of February 2020, the outbreak started in Africa. Senegal was the second sub-Saharan country to report confirmed cases of COVID-19 with an imported case detected on 2 March 2020. From March to October 2020 in Senegal, a total of 15,605 cases has been identified and 323 deaths reported [3].

However few data on COVID-19 in sub-Saharan African populations is available. In Senegal as in most countries, several debates occurred regarding care management and the use of specific treatments for COVID-19 patients. The treatment using hydroxychloroquine (HCQ) alone or a combination of HCQ and azithromycin (AZM) has been controversial. After initial publications of the potential efficacy of this regimen [4,5,6,7], a large use of this treatment was observed in many countries, including China, France, Italy, Netherlands, and Korea [8]. Some in vitro studies have shown that CQ and HCQ possess antiviral activities against many viruses, including influenza viruses and arboviruses [9]. Some authors have reported that QC/HCQ significantly inhibits virus entry and at least five steps in the replication of SARS-CoV-2 [10]. Significant effect was observed on duration of fever, duration of cough, clinical recovery, death and/or transfer to intensive care [11]. Although AZM activity against several viruses has been studied in vitro and in clinical trials [12]. Recently, the combination of HCQ and AZM has been shown to have a synergistic effect in vitro on SARS-CoV-2 and, when administered over a period of at least three days, to improve the clinical condition of patients [13]. However, other studies including clinical trials showed no effect for this treatment on hospitalized patients with COVID-19 in terms of overall mortality, initiation of ventilation, or duration of hospital stay [14,15,16,17,18]. Moreover, concerns regarding safety and especially occurrence of cardiac rhythmic disorders have been raised after administration of HCQ [19,20] and Word Health Organization (WHO) edited a recommendation against the use of HCQ in patients with COVID-19 regardless of disease severity [21]. However, the use of HCQ for COVID-19 treatment has been maintained in many countries [22].

In Senegal, case management for COVID-19 was based on symptomatic treatment at the discretion of the treating clinician. At the beginning of the epidemic, with the debate about the use of HCQ, the first patients were treated only symptomatically. In mid-March, HCQ was used by several practitioners and in early April HCQ was combined with AZM.

Here, we described the clinical outcome of patients hospitalized in five Isolation and Treatment Centres (ITC) in Senegal between 2 March 2020 and 31 October 2020. We analysed outcomes associated with the clinical status of patients within 15 days after ITC admission.

## 2. Methods

### 2.1. Study Design

We put in place a retrospective data analysis of medical records of patients, with a SARS-CoV-2 confirmed infection who were admitted in five ITCs. A care protocol was adapted from the International Severe Acute Respiratory and emerging Infections Consortium (ISARIC)/WHO Clinical Characterisation Protocol (CCP) v3.1 study [23,24].

### 2.2. Setting

In Dakar, the study was implemented in the ITCs of Fann Hospital, Dalal Jamm Hospital, and Hospital Principal of Dakar. All these three ITCs included an Intensive Care Unit. Two other ITCs were involved: one in the Diamniadio Children Hospital and the other corresponding to the Darou Marnane ITC, a community health care in the city of Touba, Diourbel region. All centres used the same protocol but the only difference between them was the availability of Intensive Care unit (ICU).

Data were collected on patients hospitalized from 2 March 2020 to 31 October 2020. According to the national strategy implement by the Senegalese Ministry of health for COVID-19 confirmed cases, after the 1st positive detection of the virus by RT-PCR, SARS-CoV-2 molecular detection was performed through nasopharyngeal and oropharyngeal swabs every 48–72 h until negativity. Hospital discharge was allowed in case of two consecutive negative RT-PCRs. The commercial LightMix Wuhan CoV E-gene and LightMix Modular Wuhan CoV RdRP-gene kits from TIB MOLBIOL (Berlin, Germany) were used for the SARS-CoV-2 qRT-PCR diagnostic tests [25]. A sample is positive if the cycle threshold (Ct) is lower or equal to 36.

### 2.3. Participants

Patients with a COVID-19 infection confirmed by RT-PCR were included in the cohort survey. Given the high number of patients exposed to the combination of HCQ-AZM, we excluded from analysis patients less than 18 years-old to avoid bias regarding indications of treatment. Due to a short-term use of treatment with HCQ without AZM, we also excluded from analysis patients receiving HCQ only.

Patients who did not receive a combination of HCQ and AZM for at least 3 days corresponding to AZM treatment duration were excluded. Finally, to avoid the impact of late treatment initiation on Clinical status at D15, we excluded patients who received a combination of HCQ and AZM before ITC admission. After admission in ITC, patients who stayed more than 3 days before being treated with HCQ and AZM were also excluded.

Patients were not treated with HCQ and AZM if they were children or had a known allergy to any of the products or had any other known contra indication for treatment, including retinopathy, QT prolongation, cardiac rhythm disorder and if they were pregnant or breastfeeding. At the beginning of the epidemic, patients were not treated with HCQ, only symptomatic treatment was recommended by the local authorities and then HCQ was introduced for a few weeks. Then they decided to treat all patients with HCQ + AZM.

### 2.4. Data Sources

Clinical data were collected from patients’ medical records. Data collected included patients’ sociodemographic details, medical history in terms of comorbidities and clinical symptoms and medication administration. Additionally, intensive care unit (ICU) status and ventilator use were reported.

A dedicated paper Case Report Form (CRF) adapted from ISARIC nCoV CRF v1.2 (Oxford, UK) [26] was filled at site level. Electronic anonymized data entry was secondarily performed using an instance of the Research Electronic Data Capture system (REDCap, Nashville, TN, USA) [27].

### 2.5. Variables Assessed

Given the high number of COVID-19 patient exposed to HCQ-AZM combination during the survey, we distinguished patients exposed or not to this treatment regimen. The HCQ + AZM group was defined as patients who have received HCQ and AZM concomitantly for at least 72 h. AZM was administered once a day at a dose of 500 mg per administration, during 3 days. HCQ was administered three times a day at a dose of 200 mg per administration, during 10 days. The group that neither received HCQ alone nor HCQ-AZM combination was defined as the control group (patients who have not received HCQ concomitantly or not with AZM). For the safety analysis, side effects potentially linked to the HCQ plus AZM regimen were self-declared by the clinician in charge of the patient.

### 2.6. Illness Severity

The illness severity of COVID-19 was defined by the National Early Warning Score (NEWS Score) version 2 [28].

### 2.7. End Point

Clinical status at Day 15 defined by discharge alive within 15 days after ITC admission versus dead or still hospitalized was considered.

### 2.8. Statistical Analysis

Continuous and categorical variables were presented as median, interquartile range (IQR) and as frequencies and percentages, respectively. We compared categorical variables using Fisher’s exact test and continuous variables using ANOVA. To explore the risk factors associated with clinical status at Days 15, unadjusted and covariate adjusted multivariate logistic models were performed. All covariates included in the multivariate models were chosen for their clinical relevance. Collinearity analysis has been performed. We conducted all analyses with R software, version 4.0.1 (Vienna, Austria) [29].

### 2.9. Ethical Aspects

This study was approved by the Senegalese National Ethics Committee for Research in Health (reference number 00000068/MSAS/CNERS/Sec, accessed on 10 April 2020).

## 3. Results

### 3.1. Characteristics of Patients

**Figure 1:**
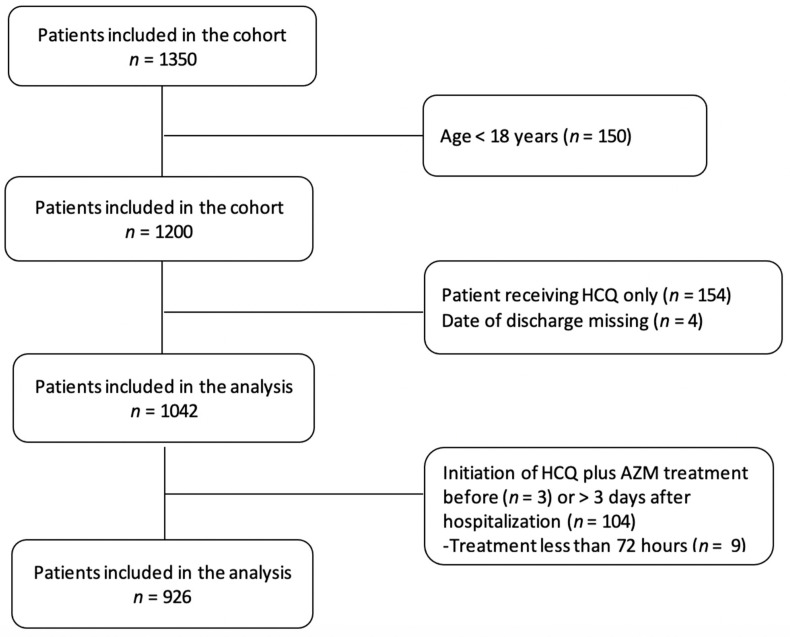
Flowchart of participants included in the Cohort study. HCQ: hydroxychloroquine; AZM: azithromycin.

A total of 1350 patients with a confirmed COVID-19 infection and admitted between 2 March and 31 October 2020 have been included. Patients less than 18 years of age, those receiving HCQ only, those with a missing date of discharge, and those who received a combination of HCQ and AZM before admission to the hospital were excluded. Patients stayed more than three days in hospital before being treated were also excluded from the analysis. Overall, 424 patients were excluded from this study. A total of 926 patients were included in the analysis (Figure 1).

### 3.2. Characteristics of Patient at ITC Admission Time

Patients’ median (IQR) age was 45 years (31–60) (Table 1). A total of 412 (44.4%) were female. The main coexisting illnesses reported were hypertension, diabetes and chronic lung disease in 155 (17.1%), 132 (14.5%) and 73 (8%) patients respectively. At least one pre-existing disorder was recorded at admission time in 33.6% of patients.

On admission, 250 (27%) of patients presented no signs of COVID-19 infection. Among the 676 patients who presented COVID-19 symptoms, fever was the most observed symptoms for 377 (55.8%), followed by cough for 322 (47.6%), myalgia for 211 (31.2%) and loss of taste or smell affected 140 (20.7%) patients. The degree of severity of COVID-19 was categorized as low in 656 (70.2%) patients. The combination of HCQ and AZM was initiated within 72 h after admission for 674 (72.8%) patients. On admission, 881 (95.1%) patients presented a positive SARS-CoV-2 PCR result. Patients without a positive PCR at baseline had a positive PCR later on were mainly suspect cases.

### 3.3. Patients’ Characteristics during Hospitalization

Median duration of hospitalization was 11 days (IQR: 8–16) (Table S1). Only 326 (35.2%) patients were categorized as low clinical risk following the NEWS score. Oxygen supplementation was required for 269 (29%) patients and 125 (13.4%) needed intensive care. Among the studied population, 91 (9.8%) deaths occurred.

Exposition to at least one antibiotic was observed for 807 (87.2%) patients. Macrolides were the most widely administered antibiotics (80.6%) followed by 3rd generation of cephalosporin (16.7%) of patients exposed.

### 3.4. Clinical Status at Day 15

Globally, 565 (61%) patients were discharged alive from the ITC within the 15 days after their admission.

In univariate analysis, treatment with combination of HCQ and AZM and absence of pre-existing disorder were significantly associated with a higher proportion of patients discharged alive within 15 days after their admission (both *p* < 0.001) (Table S2).

Patients older than 60 years (*p* < 0.001) were significantly associated with a lower proportion of discharge before 15 days after admission. Regarding symptoms at ICT admission time, absence of COVID symptoms (*p* = 0.012) was significantly associated with a higher proportion of patients discharged alive whereas cough (*p* = 0.012) and a NEWS gravity score higher than “low level” class were significantly associated with a higher proportion of patients still hospitalized or dead 15 days after their admission.

In the multivariate analysis, the treatment regimen with HCQ-AZM combination (OR: 1.63, IC 95% (1.10–2.42), *p* = 0.016) and the delay between hospitalization and symptoms onset (OR: 1.05, IC 95% (1.00–1.10), *p* = 0.042) were both significantly associated with a higher proportion of patients discharged within 15 days after their admission to ITC.

**Figure 2:**
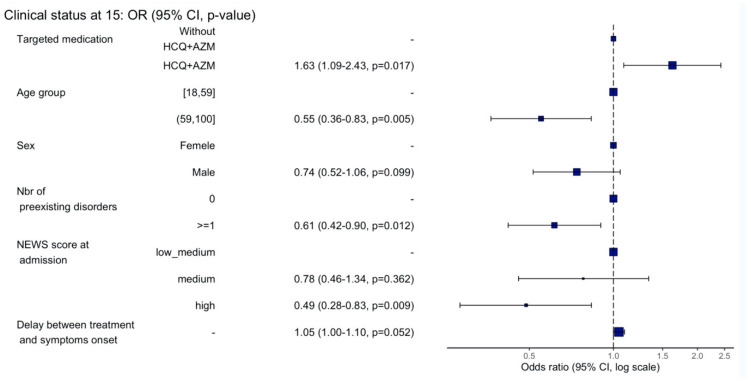
Multivariate analysis of clinical status at D15.

Older age (OR: 0.55 IC 95% (0.36–0.83), *p* = 0.004), existence of at least one comorbidity (OR: 0.59, IC 95% (0.40–0.86), *p* = 0.006) and a high NEWS severity score at admission (OR: 0.50, IC 95% (0.29–0.85), *p* = 0.011) were significantly associated with a higher proportion of patients dead or still hospitalized 15 days after their admission to ITC (Figure 2).

### 3.5. Reported Side Effects

A total of 395 side effects were reported. We observed that 91 (23%) deaths, 82 (20.7%) headache, and muscular skeletal disorders 72 (18.2%) were the most common side effects. Overall, 15 cardiac rhythm disorders were observed, 2 (13.3%) in the HCQ and AZM group and 13 (87.7%) in the without HCQ + AZM group (Table 2). Cardiac rhythm disorders observed in the HCQ + AZM group were: QT prolongation and bradycardia requiring stopping the treatment with no sequelae.

## 4. Discussion

As of today, few data are available regarding clinical and virological outcomes of COVID-19 patients living in sub-Saharan Africa. We exposed results of this first descriptive cohort in Senegal.

The population hospitalized, from March to October for COVID-19 infection described in this cohort, was younger and with less comorbidities compared to the population previously reported in Europe or the United States [30,31]. Among patients with pre-existing disorders, hypertension and diabetes were the most common. Regarding clinical presentation, the majority of patients presented a low level of severity at admission and the number of patients without any COVID-19 symptoms was high. When symptoms were present, fever, cough and myalgia were the most frequent as commonly described [32]. The political measures taken by the Senegalese government immediately after the first cases of COVID-19, and which were based on a systematic screening and testing of contact cases as well as a systematic hospitalization of all positive cases regardless of symptom presentation, could explain the higher proportion of low severity clinical presentation in hospitalized patients at the time of admission.

Patients hospitalized at an early stage of COVID-19 developed secondary symptoms, explaining the higher proportion of patients categorized as medium or high clinical risk. Complications related to the hospitalization and isolation could also explain part of this increase.

Exposure to antibiotics was extremely high due to the large prescription of macrolides. This broad prescription may have contributed to the prevention of the occurrence of infectious complications [33].

In our cohort, 61% of patients were discharged alive within 15 days after their admission. This proportion was lower than described in the multicentre, randomized, open-label, conducted in Brazil [15]. This study showed no difference in terms of improvement of clinical status at 15 days between standard care and standard care plus combination of HCQ and AZM. Hospital duration depends on criteria for admission and discharge [34]. In Senegal, following national guidelines, hospital discharge was allowed only after obtaining two consecutive negative PCRs. Moreover, recruitment of our cohort was conducted in five sites including three specialized treatment centres which included intensive care units. This specificity contributes to a higher level of severity of hospitalized patients and a higher rate of death.

A majority of the described patients (72.4%) received a combination of HCQ and AZM within 72 h following their admission to the ITC. This cohort’s results showed, in multivariate analysis, a higher proportion of patients discharged alive for patients taking combination of HCQ and AZM regimen early after their admission. However, another study in Africa confirms this finding with the combination of chloroquine and azithromycin (CQ/AZM). It shows that its patients who received CQ/AZ had significantly lower mortality than those on other treatments [35].

These results, while consistent with some studies [5,6,35,36,37], are in contradiction with clinical trials and other large-scale studies on efficacy of HCQ with or without AZM which were conducted in high income countries [15,17,18,38]. Treatment initiation at an early stage of the disease, age of the population, local hospital discharge indications may contribute to this result.

Few side effects were documented in our study including only two cases of cardiac rhythm disorder in the HCQ and AZM group. A study in Africa where the majority of patients (*n* = 630) were treated with CQ/AZ reported that eighteen out of 545 patients on CQ/AZ reported at least one side effect, which was mostly pruritus, rash, gastrointestinal disturbances, palpitations or bradycardia [35]. Chloroquine is the most effective and widely used drug in many parts of the world, and particularly in Africa, as a reliable treatment for uncomplicated forms of malaria [39]. It was a frequently kept medicine at home and used for self-treatment in most African countries. Between 1995 and 2003, chloroquine was the only drug recommended by the Senegalese health authorities [40]. Moreover, a prospective longitudinal study carried out in a Senegalese village between 1990 and 2012 reported few serious side effects linked to chloroquine [41]. Furthermore, the spread of chloroquine resistance in *Plasmodium falciparum* has had a considerable impact on the high level of malaria mortality in most epidemiological settings in Africa [42]. Hydroxychloroquine (HCQ) is a less toxic metabolite of chloroquine [43]. Most toxic effects occur at high concentrations, after prolonged administration of the drug or in combination with other drugs [44].

Delay between first symptoms onset and treatment was also correlated to a higher proportion of patients discharged at D15. This result may be related to the national recommendations for hospital discharge which was conditioned by a PCR negativity. Thus, the negativity of the nasopharyngeal swab could be obtained earlier for patients who developed their symptoms far from admission.

We showed a significantly higher proportion of patients dead or still hospitalized after 15 days post-admission among older patients, patients with at least one reported pre-existing disorder and patients categorized as high clinical risk at admission time following NEWS score. These results were in line with previously reported data [32,45,46]. The number of hospitalised cases depends more on the availability of resources and diagnostic capacity. In many African countries, due to resource limitations, the number of hospitalised cases appears to be insignificant, in contrast to the hospitalised numbers in Europe during this period. It also depends on different case detection and management policy established by the health authorities.

### Limitations of the Study

Our study has some limitations. First, some cases had incomplete documentation of the disease history before hospitalization, daily clinical symptoms and laboratory testing. Data generation was clinically driven and not systematic. Second, recruitment was performed in five ITC including three specialized treatment centres which included intensive care units. This aspect may have an overrepresentation of severe cases.

Combination of HCQ-AZM was positively correlated with an increase in the proportion of patients discharged alive at 15 days. However, further prospective trials are needed to examine this impact. Future analyses will allow more detailed description of the biological, virological and immunological profiles of the patients.

## 5. Conclusions

Our results also need to be confirmed by randomised controlled trials that strictly evaluate the efficacy of HCQ + AMZ treatment for COVID-19 in hospitalised patients in Africa. Our findings also suggest that HCQ + AMZ treatment may play an important role in reducing the length of hospital stay.

## Figures and Tables

**Table 1:** Patients’ characteristics at the time of admission in the Isolation and Treatment Centre.

Patient’s Characteristics	Without HCQ + AZM (*n* = 252)	HCQ + AZM (*n* = 674)	Total (*n* = 926)	*p*-Value
Age				<0.001 ^1^
Median	57	42	45	
Q1, Q3	36, 67	30, 58	31, 60	
Age categories				<0.001 ^2^
18–59	140 (55.6%)	521 (77.3%)	661 (71.4%)	
≥60	112 (44.4%)	153 (22.7%)	265 (28.6%)	
Sex				0.824 ^2^
Female	114 (45.2%)	298 (44.2%)	412 (44.5%)	
Number of preexisting disorders				<0.001 ^2^
0	118 (46.8%)	464 (68.8%)	582 (62.9%)	
1	68 (27.0%)	124 (18.4%)	192 (20.7%)	
≥2	66 (26.2%)	86 (12.8%)	152 (16.4%)	
Preexisting disorder				
Hypertension	72 (30.1%)	83 (12.5%)	155 (17.1%)	<0.001 ^2^
Diabetes	58 (23.8%)	74 (11.1%)	132 (14.5%)	<0.001 ^2^
Chronic lung disease *	4 (1.6%)	15 (2.2%)	19 (2.1%)	0.794 ^2^
Obesity	17 (7.1%)	21 (3.2%)	38 (4.2%)	0.014 ^2^
Chronic Heart dysfunction	13 (5.2%)	15 (2.2%)	28 (3.0%)	0.028 ^2^
Chronic hematologic dysfunction	3 (1.2%)	7 (1.0%)	10 (1.1%)	0.736 ^2^
Chronic renal disease	4 (1.6%)	1 (0.1%)	5 (0.5%)	0.020 ^2^
Others	34 (13.7%)	66 (9.9%)	100 (10.9%)	0.121 ^2^
Symptoms				
No COVID symptoms	37 (14.7%)	213 (32.2%)	250 (27.4%)	<0.001 ^2^
Fever	116 (49.2%)	261 (39.5%)	377 (42.1%)	0.011 ^2^
Cough	115 (48.9%)	207 (31.6%)	322 (36.1%)	<0.001 ^2^
Runny nose	24 (10.3%)	72 (11.0%)	96 (10.8%)	0.902 ^2^
Taste or smell lost	33 (14.1%)	107 (16.3%)	140 (15.7%)	0.465 ^2^
Myalgia	67 (28.4%)	144 (22.0%)	211 (23.7%)	0.050 ^2^
Asthenia	47 (24.9%)	80 (13.5%)	127 (16.2%)	<0.001 ^2^
Diarrhea and/or Nausea	19 (8.7%)	44 (7.0%)	63 (7.4%)	0.373 ^2^
Median no. of days since symptom onse—median (IQR)				0.359 ^1^
Median	6	6	6	
Q1, Q3	3, 8	3, 9	3, 8	
Delay between first positive PCR and ITC admission, days—median (IQR)				0.333 ^1^
Median	3	3	3	
Q1, Q3	2, 4	2, 4	2, 4	
Systolic Blood Pressure (mmHg)				<0.001 ^1^
Median	135	130	130	
Q1, Q3	120, 150	120, 140	120, 145	
Diastolic Blood Pressure (mmHg)				0.697 ^1^
Median	80	80	80	
Q1, Q3	72, 90	77, 90	76, 90	
Heart Rate (beats/min)				<0.001 ^1^
Median	96	87	89	
Q1, Q3	84, 107	76, 98	78, 100	
Respiratory rate (breaths/min)				<0.001 ^1^
Median	24	22	22	
Q1, Q3	20, 30	20, 24	20, 26	
NEWS score at admission time				<0.001 ^2^
Low	135 (53.6%)	519 (77.0%)	654 (70.6%)	
Low medium	16 (6.3%)	60 (8.9%)	76 (8.2%)	
Medium	44 (17.5%)	48 (7.1%)	92 (9.9%)	
High	57 (22.6%)	47 (7.0%)	104 (11.2%)	
Admitted to intensive care at admission (ICU)	30 (11.9%)	10 (1.5%)	40 (4.3%)	<0.001 ^2^
Virological results at admission ^†^				
Negative	-	-	45 (4.86%)	
Positive	-	-	881 (95.14%)	
Ct values—median (IQR)	-	-	30 (23.7,33)	

HCQ: Hydroxychloroquine; AZM: Azithromycin; ^†^ first PCR results following ICT admission; * Chronic lung disease was defined as chronic obstructive pulmonary disease, asthma, or chronic bronchitis. ^1^ Anova test; ^2^ Exact Fisher test.

**Table 2:** Reported side effects in the two groups of treatment.

	Without HCQ + AZM	HCQ + AZI	Total
Abdominal discomfort	14 (4.32%)	6 (8.45%)	20 (5.06%)
Cardiac rhythm disorder	13 (4.01%)	2 (2.82%)	15 (3.80 %)
Death	67 (20.68%)	24 (33.80%)	91 (23.04%)
Diarrhea	18 (5.56%)	19 (26.76%)	37 (9.37%)
Headache	78 (24.07%)	4 (5.63%)	82 (20.76%)
Hematologic disorders	6 (1.85%)	1 (1.41%)	7 (1.77%)
Musculoskeletal disorders	70 (21.60%)	2 (2.82%)	72 (18.23%)
Upset stomach	43 (13.27%)	5 (7.04%)	48 (12.15%)
Vomit	15 (4.63%)	8 (11.27%)	23 (5.82%)
Total side effect reported	324	71	395
Absence of side effect reported	88 (9.5%)	618 (66.74%)	706 (76.24%)

## Data Availability

The data presented in this study are available on request from the corresponding author.

## Supplementary Materials

The following are available online at https://www.mdpi.com/article/10.3390/jcm10132954/s1, Table S1: Patients’ clinical and virological characteristics during hospitalization period. Table S2: Clinical status of patients at day 15, risk factors associated with hospital discharge.
