# Supplementary material for: Hydroxychloroquine and Azithromycin Treatment of Hospitalized Patients Infected with SARS-CoV-2 in Senegal from March to October 2020
Source: J Clin Med. 2021 Jun 30;10(13):2954. doi: 10.3390/jcm10132954 (PMC8269162; doi:10.3390/jcm10132954)
Supplement: Supplementary file 1 [file jcm-10-02954-s001.zip › jcm-1226440-supplementary.pdf]

Table S1. Patients' clinical and virological characteristics during hospitalization period.

	Without HCQ+AZM (N=252)	HCQ + AZM (N=674)	Total (N=926)	p value
Duration of hospitalization				0.661[1]
Median	12	11	11	
Q1, Q3	8, 16	8, 16	8, 16	
Highest NEWS score				<0.001[2]
Low	57 (23.9%)	269 (44.0%)	326 (38.4%)	
Low medium	36 (15.1%)	153 (25.0%)	189 (22.3%)	
Medium	34 (14.3%)	62 (10.1%)	96 (11.3%)	
High	111 (46.6%)	127 (20.8%)	238 (28.0%)	
Oxygen needs	127 (50.4%)	142 (21.1%)	269 (29.0%)	<0.001[2]
Intensive care unit admission (ICU)	89 (35.3%)	36 (5.3%)	125 (13.5%)	<0.001[2]
Status at the end of follow-up				<0.001[2]
Discharged alive	174 (69.0%)	640 (95.0%)	814 (87.9%)	
Transferred to another hospital	11 (4.4%)	10 (1.5%)	21 (2.3%)	
Deaths	67 (26.6%)	24 (3.6%)	91 (9.8%)	
Penicillin	8 (3.2%)	10 (1.5%)	18 (1.9%)	0.110[2]
3rd Cephalosporin	87 (34.5%)	68 (10.1%)	155 (16.7%)	<0.001[2]
Fluoroquinolone	38 (15.1%)	16 (2.4%)	54 (5.8%)	<0.001[2]
Macrolides	72 (28.6%)	674 (100.0%)	746 (80.6%)	<0.001[2]
Others antibiotics	22 (8.7%)	18 (2.7%)	40 (4.3%)	<0.001[2]
Corticosteroid	63 (25.0%)	24 (3.6%)	87 (9.4%)	<0.001[2]
Vitamins	137 (54.4%)	315 (46.7%)	452 (48.8%)	0.046[2]
Zinc	1 (0.4%)	0 (0.0%)	1 (0.1%)	0.272[2]
Anticoagulant	6 (2.4%)	9 (1.3%)	15 (1.6%)	0.255[2]

1. Linear Model ANOVA
2. Fisher's Exact Test for Count Data

Table S2: Clinical status of patients on Day 15 (Univariate analysis).

		clinical status at D15		
		hospitalized or dead	discharged	OR (univariate)
Virus targeted medication	Without HCQ+AZM	135 (53.6)	117 (46.4)	-
	HCQ + AZM	226 (33.5)	448 (66.5)	2.29 (1.70-3.07, p<0.001)
Age categories	(18,39]	124 (32.0)	263 (68.0)	-
	(40,59]	92 (33.6)	182 (66.4)	0.93 (0.67-1.30, p=0.67)
	(60,93]	145 (54.7)	120 (45.3)	0.39 (0.28-0.54, p<0.001)
Sex	Female	148 (35.9)	264 (64.1)	-
	Male	213 (41.4)	301 (58.6)	0.79 (0.61-1.03, p=0.087)
Number of preexisting disorders	0	197 (32.0)	418 (68.0)	-
	≥1	164 (52.7)	147 (47.3)	0.42 (0.32-0.56, p<0.001)
Symptoms at admission				
No COVID symptoms	No	274 (41.3)	389 (58.7)	-
	Yes	82 (32.8)	168 (67.2)	1.44 (1.07-1.97, p=0.019)

Fever	No	184 (35.5)	335 (64.5)	-
	Yes	158 (41.9)	219 (58.1)	0.76 (0.58-1.00, p=0.050)
Cough	No	197 (34.6)	372 (65.4)	-
	Yes	139 (43.2)	183 (56.8)	0.70 (0.53-0.92, p=0.012)
Runny nose	No	295 (37.2)	497 (62.8)	-
	Yes	40 (41.7)	56 (58.3)	0.83 (0.54-1.28, p=0.399)
Taste or smell lost	No	291 (38.8)	459 (61.2)	-
	Yes	44 (31.4)	96 (68.6)	1.38 (0.95-2.05, p=0.099)
Myalgia	No	247 (36.3)	434 (63.7)	-
	Yes	90 (42.7)	121 (57.3)	0.77 (0.56-1.05, p=0.095)
Asthenia	No	306 (37.5)	510 (62.5)	-
	Yes	27 (40.3)	40 (59.7)	0.89 (0.54-1.49, p=0.650)
Diarrhea	No	308 (38.0)	503 (62.0)	-
	Yes	14 (35.0)	26 (65.0)	1.14 (0.59-2.27, p=0.705)
NEWS score at admission time	low	230 (35.2)	424 (64.8)	-
	low/medium	17 (22.4)	59 (77.6)	1.88 (1.10-3.40, p=0.028)
	medium	46 (50.0)	46 (50.0)	0.54 (0.35-0.84, p=0.006)
	high	68 (65.4)	36 (34.6)	0.29 (0.18-0.44, p<0.001)
Delay between admission and symptoms onset	Mean (SD)	6.2 (5.1)	6.6 (3.9)	1.02 (0.99-1.06, p=0.228)
Delay between treatment and symptoms onset	Mean (SD)	7.8 (7.0)	7.2 (4.1)	0.98 (0.95-1.01, p=0.202)
